# New York Lancet

**Published:** 1843-04

**Authors:** 


### NEW YORK LANCET.

The *New York Lancet,* which "blazed, the comet of a season," has comet-like "shot madly from its sphere"—in other words it is defunct. "We could have better spared a"—worse journal. "The rage (if such there be) for hebdomadals will be short lived—" what think you of that now, O wise son of Ballymena! "Moon-struck" quoth-a? C.

